# Special Issue: Molecular Research on Mental Disorders 2.0

**DOI:** 10.3390/ijms26199555

**Published:** 2025-09-30

**Authors:** Magdalena Sowa-Kućma

**Affiliations:** 1 Department of Human Physiology, Faculty of Medicine, Collegium Medicum, University of Rzeszów, Al. Rejtana 16C, 35-959 Rzeszów, Poland; msowa@ur.edu.pl; 2 Centre for Innovative Research in Medical and Natural Sciences, Faculty of Medicine, Collegium Medicum, University of Rzeszów, Warzywna 1a, 35-310 Rzeszów, Poland

Mental disorders remain one of the leading causes of disability worldwide, affecting more than 970 million people and contributing substantially to the global burden of disease [1]. They not only impair quality of life and social functioning but also impose significant economic costs on healthcare systems and societies [2,3]. Depression alone is projected to become the leading cause of global disease burden by 2030, underscoring the urgency of addressing this challenge [4]. Despite decades of intensive research and therapeutic advances, including the introduction of novel antidepressants, psychotherapies, and neuromodulation techniques, a considerable proportion of patients still exhibit inadequate responses or treatment resistance [5]. This therapeutic gap reflects the incomplete understanding of the molecular, cellular, and systemic mechanisms driving psychiatric conditions.

Over the past two decades, converging evidence has pointed toward dysregulation of neuroinflammation, oxidative stress, synaptic plasticity, and neuroendocrine function as critical contributors to the onset and progression of mental disorders [6,7,8,9]. Advances in neuroimaging, genomics, and computational modeling have highlighted the interplay between genetic predispositions, environmental exposures, and lifestyle-related factors, shaping a highly heterogeneous clinical landscape [10,11,12]. Accordingly, current research efforts are increasingly directed toward uncovering biomarkers that capture this complexity and can support early diagnosis, individualized treatment, and objective monitoring of therapeutic outcomes [13,14].

The Special Issue “Molecular Research on Mental Disorders 2.0” of the International Journal of Molecular Sciences brings together six contributions (three reviews and three original research articles) that provide novel insights into the molecular underpinnings of psychiatric disorders.

One important avenue of investigation concerns the multifunctional protein α-Klotho, traditionally known for its role in aging. Growing evidence suggests that α-Klotho deficiency may contribute to depression and cognitive impairment by modulating oxidative stress, glutamatergic transmission, and neuroinflammation. The review by Pańczyszyn-Trzewik and colleagues provides a comprehensive overview of the potential of α-Klotho as a molecular link between affective and cognitive dysfunctions and highlights its promise as a biomarker of depressive disorders [15].

Another promising, yet less explored, field is the role of cellular prion protein (PrP^c^) in mood disorders. While prions are primarily associated with neurodegenerative diseases, recent findings indicate that PrP^c^ in its physiological form exerts neuroprotective effects, regulates neurotransmission, and influences sleep and biological rhythms. Chrobak et al. integrate current data showing that PrP^c^ deficiency leads to depressive-like behaviors, cognitive decline, and circadian rhythm disturbances, suggesting that this protein may emerge as a novel target in biomarker research and therapeutic strategies [15].

Continuing the neurobiological theme, Krasner et al. advance the neuronal atrophy hypothesis of mood disorders. By combining neuroimaging, molecular, and genetic evidence, they propose that neuronal atrophy (driven by alterations in neurotrophic signaling, HPA axis dysregulation, mitochondrial dysfunction, and chronic inflammation) is a key mechanism underlying depression and bipolar disorder. Their review highlights biomarker discovery, computational modeling, and precision medicine approaches as pathways toward personalized treatment in psychiatry [15].

Original research articles in this issue further enrich these insights. Vega-Rivera and colleagues investigate the combined effects of chronic variable stress and a high-fat, high-sugar cafeteria diet in a menopause model. While this combination does not intensify anxiety- or depressive-like behaviors, it markedly enhances microglial activation and neuronal c-fos expression in the hippocampus, underscoring how metabolic and hormonal factors interact with stress to drive neuroinflammatory changes during the menopausal transition [15].

Depression frequently accompanies neurodegenerative disorders, including Alzheimer’s disease (AD). Qin et al. explored this intersection using a streptozotocin-induced AD rat model and revealed that activation of indoleamine 2,3-dioxygenase (IDO) and dysregulation of the kynurenine pathway in prelimbic and infralimbic cortices contribute to depression-like behaviors. Remarkably, region-specific inhibition of IDO reversed these behaviors through distinct glial mechanisms, highlighting the therapeutic promise of targeting tryptophan metabolism [15].

A complementary review by Fułek and colleagues examines how PrP^c^ interacts with amyloid-β oligomers, triggering synaptic dysfunction via Fyn kinase signaling. This work integrates evidence linking prion biology to AD pathology and underscores shared molecular pathways between neurodegenerative and psychiatric disorders [15].

Together, these reviews and studies emphasize that oxidative stress, neuroinflammation, synaptic plasticity, and neurodegeneration form interconnected networks in the pathophysiology of psychiatric disorders. They highlight the convergence of metabolic dysregulation and protein–protein interactions as critical drivers of disease and point toward integrating molecular, imaging, and computational approaches to develop robust diagnostic and therapeutic tools [15].

In conclusion, this Special Issue gathers cutting-edge evidence on the molecular basis of mental disorders, pointing to novel mechanisms and potential biomarkers such as α-Klotho and PrP^c^, and to innovative conceptual frameworks like the neuronal atrophy hypothesis. By bridging molecular neuroscience with clinical translation, the contributions collected here open new avenues for early diagnosis, targeted therapies, and precision psychiatry. We hope that “Molecular Research on Mental Disorders 2.0” will inspire further interdisciplinary research and accelerate the translation of these insights into effective clinical interventions.
